# Cell‐Penetrating Peptide‐Based Triple Nanocomplex Enables Efficient Nuclear Gene Delivery in *Chlamydomonas reinhardtii*

**DOI:** 10.1002/bit.29019

**Published:** 2025-05-08

**Authors:** Eun Jeong Sim, Quynh‐Giao Tran, Yu Rim Lee, Trang Thi Le, Hyang Ran Yoon, Dong‐Yun Choi, Dae‐Hyun Cho, Jin‐Ho Yun, Hong Il Choi, Hee‐Sik Kim, Yong Jae Lee

**Affiliations:** ^1^ Cell Factory Research Center Korea Research Institute of Bioscience and Biotechnology (KRIBB) Daejeon Republic of Korea; ^2^ Department of Environmental Biotechnology, KRIBB School of Biotechnology University of Science and Technology (UST) Daejeon Republic of Korea; ^3^ Immunotherapy Convergence Research Center Korea Research Institute of Bioscience and Biotechnology (KRIBB) Daejeon Republic of Korea

**Keywords:** cell‐penetrating peptide, gene delivery, microalgae, nanocomplex, nuclear localization signal, pVEC, SV40

## Abstract

Microalgae are a promising solution for mitigating climate change due to their ability to capture greenhouse gases and produce renewable materials. However, their effective application is often hindered by barriers that necessitate advances in genetic engineering to improve photosynthesis and productivity. One major obstacle is the microalgal cell wall, which complicates the delivery of genetic material into these organisms. To address these challenges, we developed a novel triple nanocomplex system integrating cell‐penetrating peptides (CPPs), nuclear localization signal (NLS) peptides, and plasmid DNA. This system allows simple preparation while achieving efficient nuclear translocation of plasmid DNA. We evaluated two CPPs, pVEC‐ORI and pVEC‐R6A, for their efficacy in facilitating intracellular transfer of DNA into wild‐type *Chlamydomonas reinhardtii* cells. Notably, pVEC‐R6A demonstrated a 6.88‐fold increase in efficiency compared to pVEC‐ORI, despite the presence of thick cell walls. The optimal CPP:DNA ratio for stable nanocomplex formation was determined to be 5:1 for pVEC‐ORI and 10:1 for pVEC‐R6A. By incorporating the simian virus 40 (SV40) NLS into CPP/DNA nanocomplexes, we successfully directed the localization of plasmid DNA into the nucleus. Our findings indicate that this simple and efficient DNA delivery system has significant potential as a tool to advance microalgal synthetic biology.

## Graphical abstract

A novel strategy for nuclear gene delivery in microalgae using a cell‐penetrating peptide (CPP) and nuclear localization signal (NLS) is described. The authors developed a triple nanocomplex of CPP, plasmid DNA, and SV40 NLS to enhance nuclear delivery in Chlamydomonas reinhardtii. Fluorescence co‐localization and stability analysis revealed that pVEC‐R6A combined with SV40 efficiently delivered plasmid DNA to the nucleus and enabled successful genetic transformation.

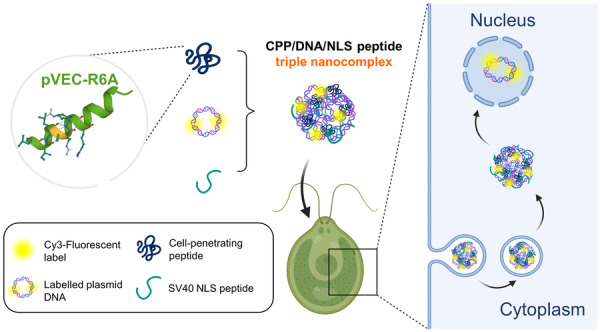

## 1 Introduction

Microalgae are photosynthetic organisms that harness sunlight to produce energy. They play a crucial role in reducing greenhouse gases while having the potential to generate high‐value end products. Microalgae production efficiency is significantly influenced by metabolic engineering efforts aimed at increasing the yield of desired metabolites. However, challenges arise due to the complexity of their metabolic pathways and issues related to genetic stability (Ranjbar and Malcata 2022). Conventional transformation methods such as electroporation, particle bombardment, and *Agrobacterium*‐mediated techniques are commonly used to deliver genetic material (Shi et al. 2021). For instance, electroporation is a common method for microalgae transformation, in which an electric pulse is applied to create temporary pores in the cell membrane, allowing exogenous molecules such as DNA and proteins to pass through. However, a major drawback is the potential to cause substantial cell death, especially in sensitive species. In contrast, *Agrobacterium*‐mediated transformation takes advantage of *Agrobacterium tumefaciens* to deliver DNA fragments into host cells through natural horizontal gene transfer. This results in stable integration into transcriptionally active regions of the genome, without causing physical damage to the cell. Despite its advantages, this method is time‐consuming and labor‐intensive (Cheng et al. 2012; Choi et al. 2022). Furthermore, biolistic transformation utilizes DNA‐coated microparticles to effectively cross cell wall barriers. However, this method requires specialized and expensive equipment and typically yields relatively low transformation efficiency compared to other methods. In addition to the potential for physical damage to host cells, which may impede successful regeneration of transformants, the biolistic approach also carries the risk of introducing high transgene copy numbers that may trigger gene silencing (Ortiz‐Matamoros et al. 2017). Therefore, the optimization of transformation techniques is critical for microalgal biotechnology advancement (Suresh and Kim 2013).

In this context, cell‐penetrating peptides (CPPs) have emerged as promising non‐cytotoxic alternatives for molecular delivery (Lee et al. 2021). These peptides are capable of efficiently delivering a wide range of molecules, including DNA, RNA, and proteins, into living cells within 20 min of simple treatment (Lindgren et al. 2000; Lundberg and Langel 2003). The applicability of CPPs extends across various organisms, including bacteria, mammals, plants, and microalgae (Kristensen et al. 2016). Notably, research indicates that the transformation efficiency of CPPs is approximately 3.5‐fold higher than that of electroporation, especially for the delivery of large plasmid DNA in *Escherichia coli* (Islam et al. 2019). The efficacy of CPPs in enhancing the internalization of molecules is attributed to various mechanisms, such as direct translocation and endocytosis, making them promising tools for transfection (Nabil et al. 2013). Given the limitations associated with conventional genetic modification methods in microalgae, CPP offers an attractive alternative to enhance the genetic transformation of microalgae by allowing direct delivery of genetic material while maintaining high cell viability after transformation.

Despite these advantages, CPP delivery efficiency varies due to differences in cell wall structure among microalgal species. For example, the R9 CPP, composed of nine arginine residues, effectively delivered double‐stranded (ds) RNA to *Dunaliella salina*, a species with a thin cell wall, but failed in species with relatively thicker cell walls, such as *Chlamydomonas reinhardtii*, *Chlorella vulgaris*, and *Phaeodactylum tricornutum* (Wei et al. 2015). Identifying versatile CPPs that can penetrate various cell wall types is essential for effective genetic engineering of microalgae. Among such candidates, pVEC has demonstrated superior ability in delivering macromolecules in *C. reinhardtii* compared to other CPPs, including R9, transportan, TAT, and PEN (Kang et al. 2017). Moreover, recent studies highlight the potential of pVECs in clustered regularly interspaced palindromic repeat (CRISPR) integration, successfully delivering ribonucleoprotein complexes for microalgal gene editing (Kang et al. 2020). The ability of pVEC to penetrate cell walls, regardless of thickness, supports its suitability for microalgal genetic engineering (Chugh and Eudes 2008; Kang et al. 2017).

Once the genetic material is internalized, directing it to the nucleus poses another challenge. Efficient nuclear targeting is essential for the successful integration of foreign genes into eukaryotic cells (Borer et al. 1989). However, naked nucleic acids encounter barriers during nuclear import due to their large size and negative charge, which hinder their passage through the cell and nuclear membranes (Escriou et al. 2003; Sokolova and Epple 2008). Nuclear localization signal peptides (NLSs), such as the simian virus 40 (SV40) peptide, function by binding to importin proteins to form a transport complex that moves through the nuclear pore complex (van der Aa et al. 2005). However, coupling NLSs to DNA does not significantly improve transfection efficiency, as NLSs mainly facilitate nuclear transfer rather than cellular uptake (Nagasaki et al. 2003; van der Aa et al. 2005). Thus, strategies combining CPPs with NLSs show promise for enhancing foreign gene transport across the nuclear membrane, thereby improving the overall efficiency of gene delivery. Previous studies have shown that fusion of SV40 peptides with polycationic CPPs improves nuclear localization (Düzgüneş et al. 2021; Wang et al. 2011). However, the efficiency of these fusion peptides (NLS‐CPP or CPP‐NLS) in mediating dsDNA import into the nucleus remains negligible (Duvshani‐Eshet et al. 2008). Such limitations arise from interference between fused peptides, which disrupts the helical structures and impairs the functionality of their termini (Kiyoshi et al. 2023). From a practical standpoint, the fusion of CPP and NLS peptides may not be suitable for industrial applications, as they require careful selection of linkers and extensive optimization using complex structural predictions.

In this study, we investigated the potential of nanocomplexes incorporating CPP, plasmid DNA, and SV40 NLS peptide to successfully deliver plasmid DNA to *C. reinhardtii* CC‐124 without relying on additional methods such as electroporation. We examined the CPP:DNA ratio in combination with SV40 NLS to confirm the formation of triple nanocomplexes using two types of CPPs: pVEC‐ORI and pVEC‐R6A. Subsequently, we assessed the localization of plasmid DNA in relation to nuclear DNA in algal cells. Transformation mediated by the triple nanocomplex was also examined in *C. reinhardtii*. Our findings suggest that the established CPP‐based triple nanocomplex approach is a promising strategy for gene delivery in microalgae.

## 2 Methods

### 2.1 Peptide Synthesis

In this study, peptides with enhanced penetration efficiency and nuclear localization in microalgae were designed. The original pVEC peptide was termed pVEC‐ORI (LLIILRRRIRKQAHAHSK), whereas the modified pVEC peptide was termed pVEC‐R6A (LLIILARRIRKQAHAHSK). All peptides were synthesized using the Fmoc‐solid‐phase peptide synthesis method with APS‐48S (Peptron, Korea). Both pVEC‐ORI and pVEC‐R6A were labeled with FITC and linked to 6‐aminohexanoic acid (Peptron, Korea). An NH_2_ group was also added to the C‐terminus of both pVEC peptides for stabilization. Rhodamine B (Peptron, Korea) was conjugated to the N‐terminus of the SV40 peptide (PKKKRKV). All peptides exhibited > 90% purity, as confirmed via high‐performance liquid chromatography by Peptron. The peptides were dissolved in nuclease‐free water (Thermo Fisher Scientific, USA) at a concentration of 1 mM, aliquoted, and stored in a deep freezer at −80°C until use.

### 2.2 Evaluation of Nanocomplex Formation Between CPPs and Plasmid DNA

The complexation of CPPs with DNA was evaluated by electrophoretic mobility shift assay (EMSA). Briefly, an appropriate volume of CPP solution (1 mM) was added to 0.5 µg of pChlamy4 vectors (3640 bp; Thermo Fisher Scientific, USA) in a final volume of 100 µL containing nuclease‐free water (Thermo Fisher Scientific, USA) to achieve various CPP:DNA ratios (0.1:1, 1:1, 5:1, 10:1, 15:1, and 20:1). The CPP:DNA ratio was determined using the molar ratio of the amine group (N) in the peptide to the phosphate group (P) in the plasmid DNA. The mixtures were incubated for 20 min at 25°C. For triple nanocomplex formation, CPP:DNA mixtures at various molar ratios were prepared as described above, and SV40 NLS peptide was added to a final concentration of 50 µM, followed by incubation for 20 min at 25°C. For EMSA analysis, 10 µL of each sample was mixed with 6× loading dye (BIOFACT, Korea), loaded onto a 1% (w/v) agarose gel, and electrophoresis was performed with Tris‐acetate‐EDTA buffer (Thermo Fisher Scientific, USA) at 100 V for 40 min. Finally, gel images were captured using a ChemiDoc MP imager (Bio‐Rad, USA).

### 2.3 Size and Zeta Potential Assessment of the CPP/DNA Nanocomplexes

Dynamic light scattering was used to determine the size and zeta potential of the CPP/DNA nanocomplexes to evaluate the nanocomplex formation. The nanocomplexes were prepared by adding appropriate amounts of CPP to 100 µL of solution containing 1 µg of pChlamy4 plasmid DNA to achieve a CPP:DNA ratio of 5:1 for pVEC‐ORI and 10:1 for pVEC‐R6A. For the preparation of the triple nanocomplex, the same amount of plasmid DNA was used, while the CPP:DNA ratio was adjusted to 2.5:1 for pVEC‐ORI and 5:1 for pVEC‐R6A based on the results from EMSA analysis. SV40 peptide was then added to this CPP/DNA mixture to a final concentration of 50 µM. Before size and zeta potential assessment, the mixtures were sonicated for 15 min and diluted 10‐fold with nuclease‐free water. After filtering through a 0.45‐μM syringe filter (Sigma‐Aldrich, USA) and vortexing for 30 s, the nanocomplex solutions were analyzed using a Zetasizer Nano ZS‐FLS10 (Malvern Panalytical, UK) at the National NANOFAB Center (Korea).

### 2.4 Microalgae Cultivation

*C. reinhardtii* CC‐124 strain was purchased from the *Chlamydomonas* Resource Center at the University of Minnesota (http://chlamycollection.org, USA). Cells were cultured in Tris‐acetate phosphate (TAP) medium (Gorman and Levine 1965) under continuous illumination of 50 µmol photons m^−^^2^ sec^−^^1^ at 25°C, with shaking at 120 rpm. 50 mL of cells in exponential growth phase were centrifuged at 2000 rpm for 5 min. Then, the cell pellet was resuspended in TAP medium at a final concentration of 1.5 × 10^7^ cells mL^−1^ and used for further experiments. Cell density was determined using a hemocytometer (INCYTO, Korea).

### 2.5 Fluorescence Microscopic Analysis

Direct penetration of CPP was monitored via confocal laser scanning microscopy (CLSM) (Carl Zeiss, Germany). Before CLSM analysis, a total of 1.5 × 10^7^ cells were harvested at 2000 rpm for 5 min, washed with nuclease‐free water to remove the culture medium, and mixed directly with 100 µL of 25 µM pVEC‐ORI or pVEC‐R6A, followed by 1 h incubation at 25°C. CLSM was used to visualize the cellular internalization of pVEC‐ORI and pVEC‐R6A at excitation/emission wavelengths of 491/516 nm for FITC detection.

FITC‐labeled pVEC and Cyanine‐3 (Cy3)‐labeled DNA were used to assess the internalization of CPP/DNA nanocomplexes in microalgal cells. Cy3‐labeled pChlamy4 plasmid DNA was prepared using the Label IT Nucleic Acid Labeling Kit (Mirus Bio LLC, USA). A volume of 100 µL of each CPP/DNA nanocomplex was prepared as described in Section 2.3 and mixed directly with the cell pellet containing approximately 1.5 × 10^7^ cells, followed by 1 h incubation at 25°C. FITC fluorescence was observed at 491/516 nm, while Cy3 fluorescence was observed at 555/560–580 nm.

To evaluate the ability of SV40 peptide to facilitate nuclear localization of CPP/DNA nanocomplexes, a volume of 100 µL of pVEC‐R6A/DNA/SV40 triple nanocomplexes were prepared as described in Section 2.3 and mixed with the cell pellet containing approximately 1.5 × 10^7^ cells, followed by 1 h incubation at 25°C. The cells were then stained with 20 μg mL^−1^ 4′,6‐diamidino‐2‐phenylindole (DAPI) solution (Thermo Fisher Scientific, USA) for 15 min to assess nuclear localization. DAPI fluorescence was observed at 405/430–515 nm. In all experiments, chlorophyll *a* fluorescence was observed at 655/667 nm and served as a reference for normalization and microalgal cell identification. Cells were washed with nuclease‐free water before being subjected to CLSM.

### 2.6 Investigation of Cellular Uptake Mechanism of Triple Nanocomplexes

To evaluate the cellular uptake mechanism of the triple nanocomplexes in microalgae, a fluorescence‐based assay was performed with wortmannin (Sigma‐Aldrich, USA) to determine the potential involvement of endocytosis in the process. A volume of 100 µL of *C. reinhardtii* cells (1.5 × 10^7^ cells mL^−1^) per well was seeded in a 96‐well plate with wortmannin at a final concentration of 33 µM. Cells treated with TAP medium containing 5% dimethyl sulfoxide were used as controls. After 1 h, the cells were collected, washed with nuclease‐free water, and incubated with the pVEC‐R6A/Cy3‐labeled DNA/SV40 nanocomplex mixture for 1 h at 25°C. Finally, the Cy3 fluorescence intensity of the samples was measured at 532 nm using a TECAN microplate reader (Bionics, Korea).

### 2.7 Genetic Transformation Using pVEC‐R6A/DNA/SV40 Triple Nanocomplex

The triple nanocomplex mixture was prepared in a total volume of 1 mL with 65.26 µM pVEC‐R6A, 50 nM DNA (to achieve a CPP:DNA ratio of 10:1 based on the N:P ratio), and 50 µM SV40. A 100 µL aliquot of the mixture was mixed with a cell pellet containing approximately 1.5 × 10^7^ cells and incubated for 1 h at 25°C for transformation. After incubation, 10 mL of TAP medium was added and the cells were allowed to recover for 16 h in the dark with gentle shaking. The cells were then centrifuged and resuspended in 100 µL of TAP medium and plated on 1% TAP agar plates containing 5 ppm zeocin. Colonies appeared after 7 days of growth under continuous illumination of 50 µmol photons m^−2^ sec^−1^ at 25°C. Colony PCR analysis was performed on randomly selected zeocin‐resistant colonies using the primer sets: forward primer CAA CAT CTT AAA ATG GCC AGG TGA G and reverse primer AGC AGG TCG AAG TTC AGG GT, targeting the zeocin resistance gene. The experiment was repeated several times (at least five biological replicates) and the average number of zeocin‐resistant colonies obtained per replicate with an initial cell number of 1.5 × 10^7^ was expressed as transformation efficiency.

### 2.8 Statistical Analysis

Data were analyzed using Student's *t*‐test, with statistical significance set at *p < 0.05, **p < 0.01, and ***p < 0.001 based on independent replicates compared to other groups.

## 3 Results and Discussion

### 3.1 Evaluation of Cellular Internalization Efficiency of CPP

**Figure 1:**
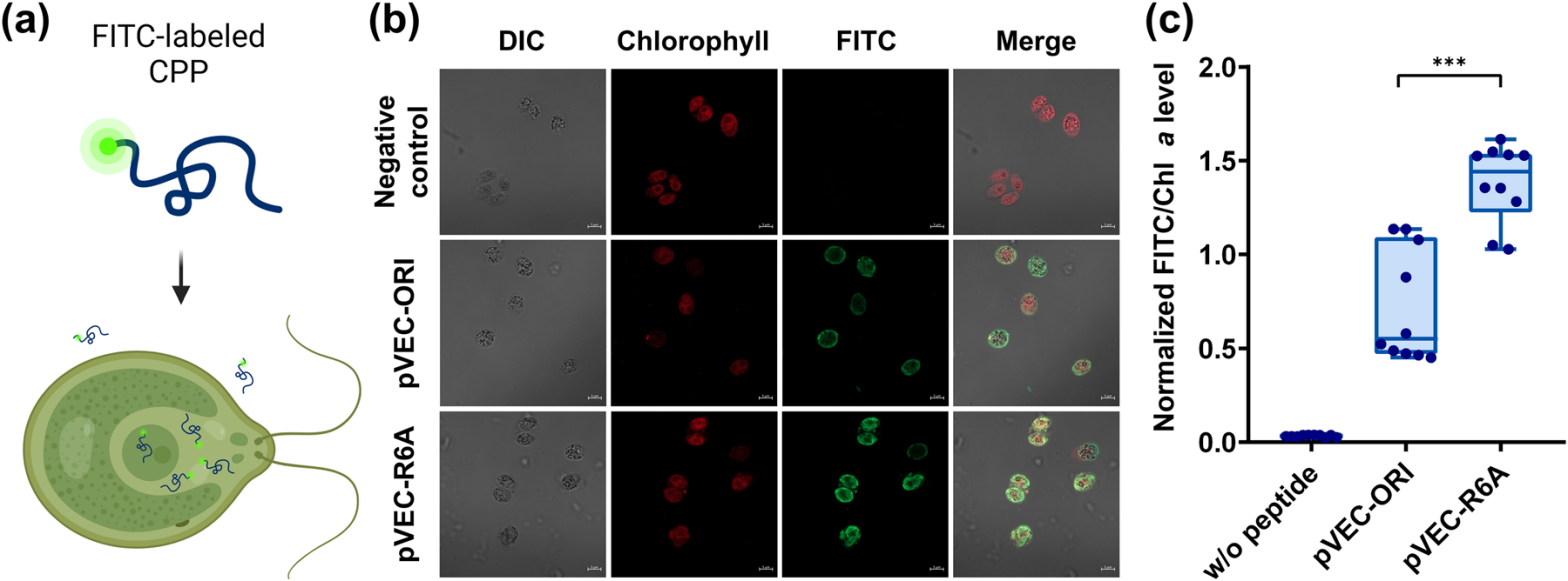
Cell penetration and translocation of cell‐penetrating peptides (CPPs) in microalgae. (a) Schematic illustration of CPP uptake by microalgal cells. (b) Confocal microscopy images showing the translocation of fluorescein isothiocyanate (FITC)‐labeled pVEC‐ORI and pVEC‐R6A peptides in *Chlamydomonas reinhardtii*. Cells were incubated with 25 μM of pVEC‐ORI and pVEC‐R6A for 1 h at 25°C. After incubation, cells were harvested by centrifugation and washed with nuclease‐free water before analysis by confocal laser scanning microscopy. Scale bar, 5 μM. (c) Fluorescence intensity of FITC in microalgal cells 1 h after treatment with 25 μM FITC‐labeled pVEC‐ORI and pVEC‐R6A (*n* = 10). Asterisks in the box plots indicate significant differences in mean values between two treatments, analyzed using Student's *t*‐test. Statistical significance is indicated by ***p < 0.001.

Cellular uptake efficiency of CPPs largely depends on their specific amino acids (Rittner et al. 2002). Some residues within CPPs play key roles in determining their ability to interact with the cell membrane and access intracellular targets (Derossi et al. 1996). Considering the distinct structural characteristics of microalgal cell walls, the selection of CPPs and their residues is crucial for the efficient delivery of cargo molecules (Wei et al. 2015). In this study, we used the amphipathic pVEC peptide, which is an effective tool for macromolecule delivery to microalgal cells (Elmquist et al. 2001; Oehlke et al. 1998; Scheller et al. 1999; Suresh and Kim 2013). Structure–activity relationship analysis of pVEC has shown that substituting the sixth arginine with l‐alanine enhances its uptake by human Bowes melanoma cells (Elmquist et al. 2006). Therefore, we designed a modified peptide, pVEC‐R6A, to improve the delivery efficiency in microalgal cells. To confirm cellular uptake, FITC was attached to the N‐terminus of pVEC‐ORI and pVEC‐R6A, and labeled CPPs were applied to microalgal cells (Figure 1a). Fluorescence was distributed throughout the cytoplasm, without any preferential localization (Figure 1b). Normalized FITC/chlorophyll *a* fluorescence intensity in the cytosol was 0.720 ± 0.300 for pVEC‐ORI and 1.381 ± 0.209 for pVEC‐R6A after treatment with the same concentration of FITC‐labeled peptides (Figure 1c). Notably, the intensity of pVEC‐R6A was 1.9‐fold higher than that of pVEC‐ORI. The increase in hydrophobic regions by the incorporation of alanine at position 6 enhanced cellular uptake in microalgae, which is analogous to the results observed in human Bowes melanoma cells (Elmquist et al. 2006). CPPs with improved cellular penetration efficiency are more effective in delivering proteins, such as insulin, to the intestines of male Sprague–Dawley rats (Kamei et al. 2013). Furthermore, nucleic acid delivery to plant cells is significantly improved using CPPs with superior penetration efficiency (Thagun et al. 2022). These findings underscore the importance of comparing uptake efficiencies to select the most suitable CPPs for cargo delivery. In this study, pVEC‐R6A exhibited high uptake efficiency and translocation across microalgal cell walls. Moreover, modifications of amino acid residues significantly improved the peptide penetration efficiency in *C. reinhardtii* CC‐124.

### 3.2 Evaluation of Nanocomplex Formation Efficiency Between CPPs and DNA

**Figure 2:**
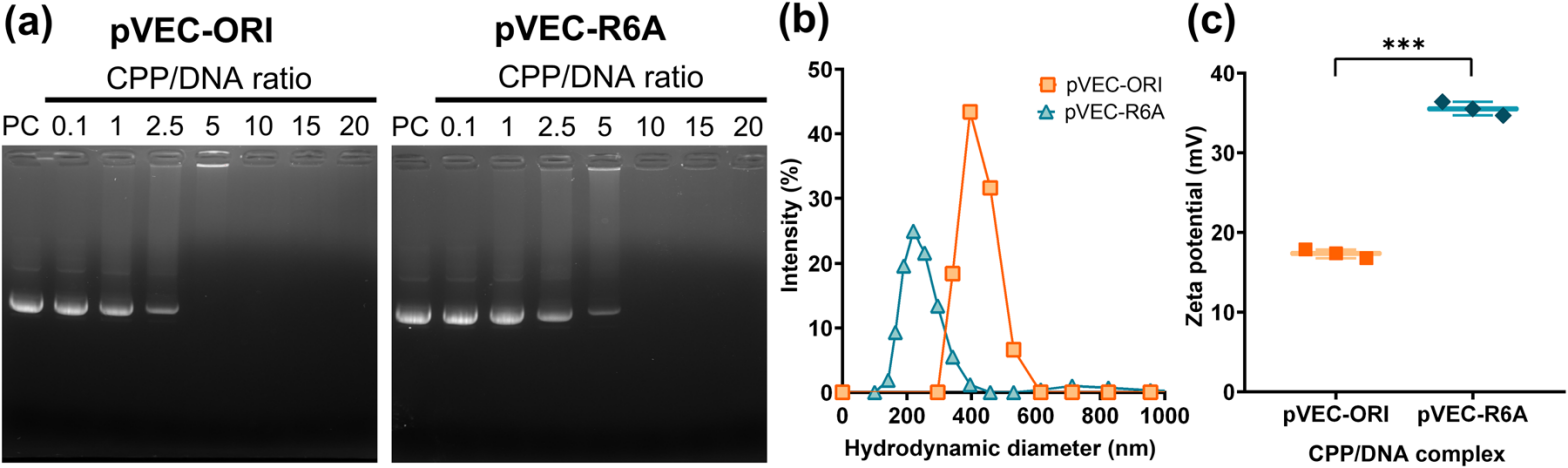
Formation and characterization of CPP/DNA nanocomplex. (a) Agarose gel electrophoresis of CPP/DNA nanocomplexes. 0.5 μg of pChlamy4 plasmid DNA (3640 bp) was mixed with pVEC‐ORI and pVEC‐R6A at varying CPP:DNA ratios (0.1:1, 1:1, 2.5:1, 5:1, 10:1, 15:1, and 20:1). Positive control (PC) consisted of naked plasmids. (b) Size and (c) zeta potential of CPP/DNA nanocomplexes measured at CPP:DNA ratios of 5:1 for pVEC‐ORI and 10:1 for pVEC‐R6A using a Zetasizer. Statistical significance is indicated by ***p < 0.001 (Student's *t*‐test).

CPPs are used for cargo delivery in two main strategies. The first approach uses chemical linkers to form covalent bonds between CPPs and their cargo, whereas the second approach relies on electrostatic self‐assembly to generate noncovalent complexes (Silhol et al. 2002). In this study, cationic CPPs were designed to interact with anionic plasmid DNA to form noncovalent complexes. The CPP:DNA ratio is critical, as complexes are formed via electrostatic interactions between the amine groups of the peptide and the phosphate groups of the DNA (Chen et al. 2007; Mo et al. 2012; Zhao et al. 2009). Furthermore, determining the optimal CPP:DNA ratio is essential to ensure cell membrane passage via the energy‐dependent endocytic pathway (van Asbeck et al. 2013). EMSA was used to determine the optimal CPP:DNA ratio for pVEC‐ORI and pVEC‐R6A. As a rapid and sensitive technique, EMSA detects protein–nucleic acid interactions based on the principle that protein–nucleic acid complexes migrate more slowly than their free nucleic acid counterparts (Hellman and Fried 2007). The optimal CPP:DNA ratio for pVEC‐ORI was found to be 5:1, indicating the point of complete binding, as confirmed by the absence of the corresponding free DNA band (Figure 2a). A higher ratio of 10:1 was required for pVEC‐R6A to achieve similar binding efficiency (Figure 2a).

Subsequently, the particle sizes of the nanocomplexes were evaluated, and the highest intensity was observed at 220 nm for pVEC‐R6A (24.9%) and 396 nm for pVEC‐ORI (43.4%) (Figure 2b). The particle size of pVEC‐R6A was reduced by 44.4% compared to that of pVEC‐ORI. Zeta potential, which represents the electrical properties of the nanoparticle surface, is influenced by factors such as particle size, synthesis method, and medium pH (Webster et al. 2005). To evaluate the surface electrical properties of the nanocomplexes, zeta potentials were measured. The zeta potentials were 17.4 ± 0.55 mV and 35.5 ± 0.85 mV for pVEC‐ORI and pVEC‐R6A, respectively (Figure 2c). Notably, the zeta potential of pVEC‐R6A was 2.04‐fold higher than that of pVEC‐ORI. A zeta potential ≥ ±30 mV is considered necessary to maintain stability in aqueous dispersions through electrostatic interactions (van Nieuwenhuyzen and Szuhaj 1998). Thus, pVEC‐R6A displayed more sustained stability in aqueous dispersions compared to pVEC‐ORI. Collectively, these results suggest that the modified CPPs form stable nanocomplexes with DNA in colloidal solutions.

### 3.3 Translocation of CPP/DNA Nanocomplexes to the Cytosol in Microalgae

**Figure 3:**
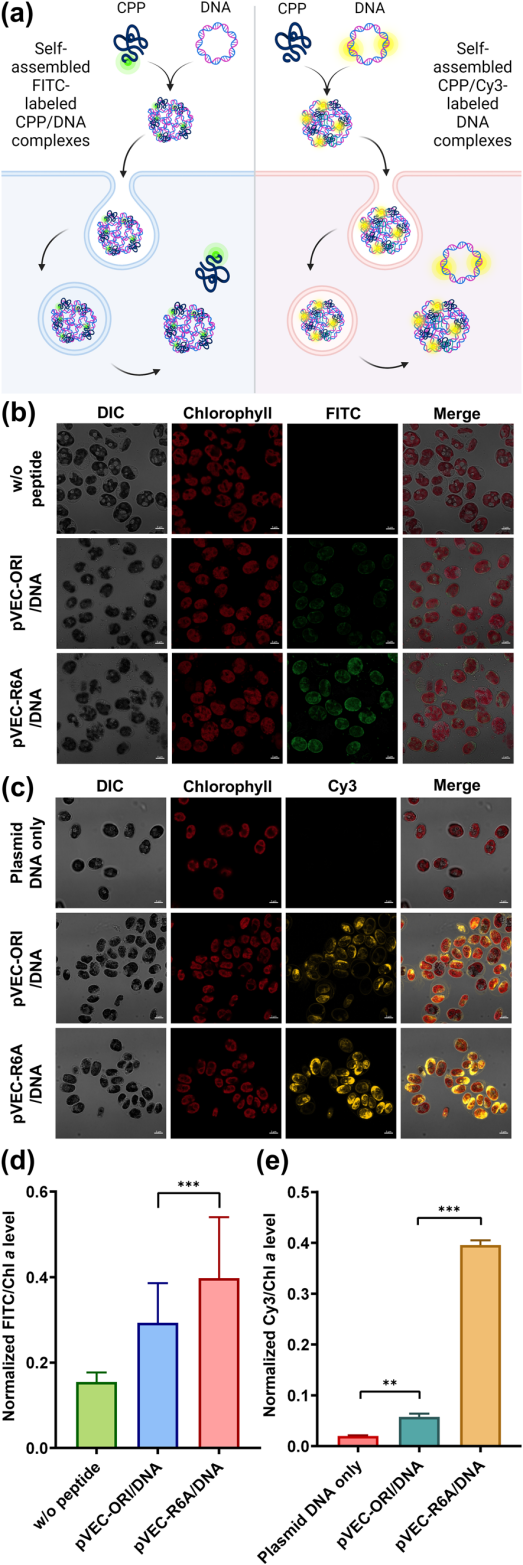
Translocation of CPP/DNA nanocomplexes in microalgal cells. (a) Proposed mechanism of cellular uptake of FITC‐labeled CPP/DNA nanocomplexes and CPP/Cy3‐labeled DNA nanocomplexes in microalgae. (b) Cellular internalization of FITC‐labeled pVEC‐ORI and pVEC‐R6A in complex with plasmid DNA at CPP:DNA ratios of 5:1 and 10:1, respectively. (c) Cellular internalization of Cy3‐labeled plasmid DNA in free form or in complex with CPP, analyzed via confocal laser scanning microscopy. Fluorescence intensity was measured at 516 nm for FITC (d) and 560–580 nm for Cy3 (e). Statistical significance is indicated by **p < 0.01 and ***p < 0.001 (Student's *t*‐test).

After the formation of stable nanocomplexes between CPP and plasmid DNA, their translocation efficiency was evaluated by introducing these nanocomplexes into microalgal cells. Their delivery was confirmed using FITC‐labeled CPPs (Figure 3a, left side). Both pVEC‐ORI and pVEC‐R6A nanocomplexes exhibited significant FITC fluorescence compared to the control group, confirming successful translocation (Figure 3b). Further quantitative analysis revealed that FITC fluorescence in cells treated with pVEC‐R6A nanocomplex was 1.35‐fold higher than that in cells treated with pVEC‐ORI nanocomplex (Figure 3d). Following the validation of peptide delivery via FITC, the translocation of plasmid DNA to the cytosol was confirmed using Cy3‐labeled DNA (Figure 3a, right side, and 3c). The normalized Cy3/chlorophyll *a* fluorescence intensity of the pVEC‐ORI nanocomplex was 2.87‐fold higher than that of the control, indicating its efficient DNA transport ability (Figure 3e). Moreover, the Cy3 intensity of the pVEC‐R6A nanocomplex was 19.75‐fold higher than that of the control and 6.88‐fold higher than that of the pVEC‐ORI nanocomplex (Figure 3e). These results highlight the superior plasmid DNA delivery efficiency of the pVEC‐R6A nanocomplex compared to pVEC‐ORI. The difference in nanocomplex properties may be responsible for the different abilities of pVEC‐ORI and pVEC‐R6A nanocomplexes in delivering plasmid DNA (Figure 2).

### 3.4 Formation of CPP/DNA/SV40 Triple Nanocomplex

**Figure 4:**
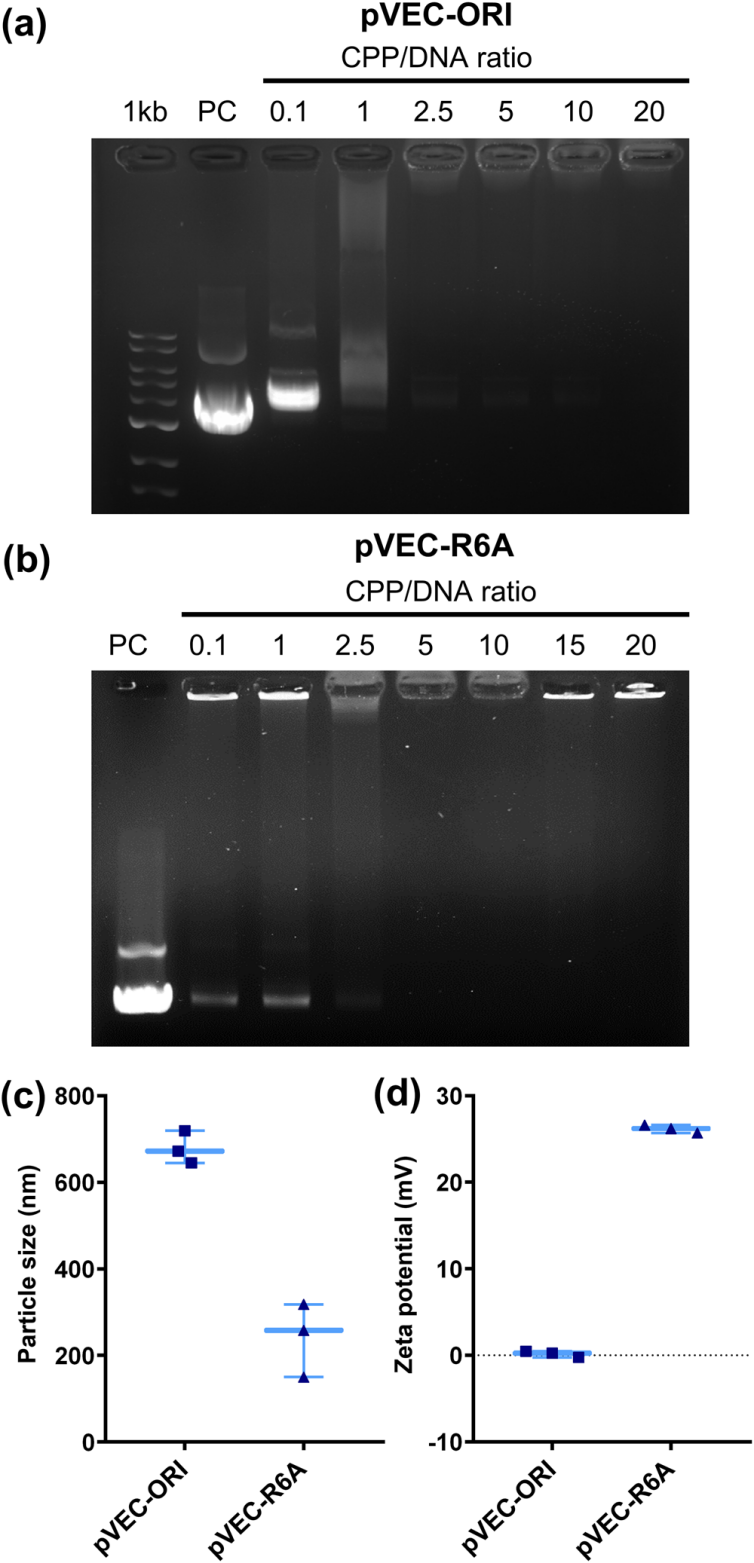
Formation and characterization of triple nanocomplex. Confirmation of triple nanocomplex formation between (a) pVEC‐ORI/DNA/SV40 and (b) pVEC‐R6A/DNA/SV40. The CPP:DNA ratios used were consistent with those previously tested for the formation of CPP/DNA nanocomplexes. SV40 peptide was added at a concentration of 50 μM. Positive control (PC) consisted of naked plasmids. (c) Size and (d) zeta potential of the triple nanocomplexes were measured at CPP:DNA ratios of 2.5:1 for pVEC‐ORI and 5:1 for pVEC‐R6A using a Zetasizer.

Enhanced cellular uptake and nuclear localization are essential for efficient gene delivery. In this study, we used the SV40 NLS peptide, a signal peptide that interacts with importin alpha, to facilitate the nuclear transport of CPP/DNA nanocomplexes. First, the optimal conditions for the formation of triple nanocomplexes were determined by EMSA, with the expectation that, upon electrophoresis, the disappearance of free DNA bands would indicate efficient complex formation (Hellman and Fried 2007). To achieve this, the CPP concentration was varied while the plasmid DNA and SV40 concentrations were kept constant. Successful formation of a triple nanocomplex between pVEC‐ORI, SV40, and plasmid DNA was achieved when pVEC‐ORI was added at a final CPP:DNA ratio of 2.5:1 (Figure 4a). On the other hand, pVEC‐R6A formed triple nanocomplexes at a CPP:DNA ratio of 5:1 (Figure 4b).

Next, the particle size and zeta potential of the triple nanocomplexes were evaluated. The particle size of the pVEC‐ORI/DNA/SV40 nanocomplex was 678.7 ± 37.4 nm, while that of the pVEC‐R6A/DNA/SV40 nanocomplex was 242 ± 85.1 nm (Figure 4c). Particle size comparison showed that pVEC‐R6A exhibited a 64.3% reduction in size compared to pVEC‐ORI. The zeta potentials of pVEC‐ORI and pVEC‐R6A were 0.16 ± 0.37 and 26.2 ± 0.45 mV, respectively (Figure 4d). Notably, the zeta potential of pVEC‐R6A was approximately 163‐fold higher than that of pVEC‐ORI. Typically, lower zeta potential values indicate increased instability and a higher risk of aggregation in colloidal solutions (Heurtault 2003). Upon aggregation, the nanocomplex size becomes unstable, increasing the difficulty of intracellular access (Ife et al. 2018). With the incorporation of SV40, pVEC‐R6A demonstrated both smaller particle size and higher zeta potential than pVEC‐ORI, thereby forming a more stable complex under colloidal conditions. Compared with previous evaluations of CPP and DNA nanocomplexes (Figure 2), the SV40 peptide showed an enhanced ability to condense plasmid DNA in triple nanocomplexes with pVEC‐R6A. Owing to its cationic nature, the SV40 peptide exerts a synergistic effect with CPP on plasmid DNA. These results suggest that the triple nanocomplex system consisting of CPP, DNA, and NLS is suitable for DNA delivery in microalgae.

### 3.5 Nuclear Localization With CPP/DNA/SV40 Triple Nanocomplex for Gene Delivery

**Figure 5:**
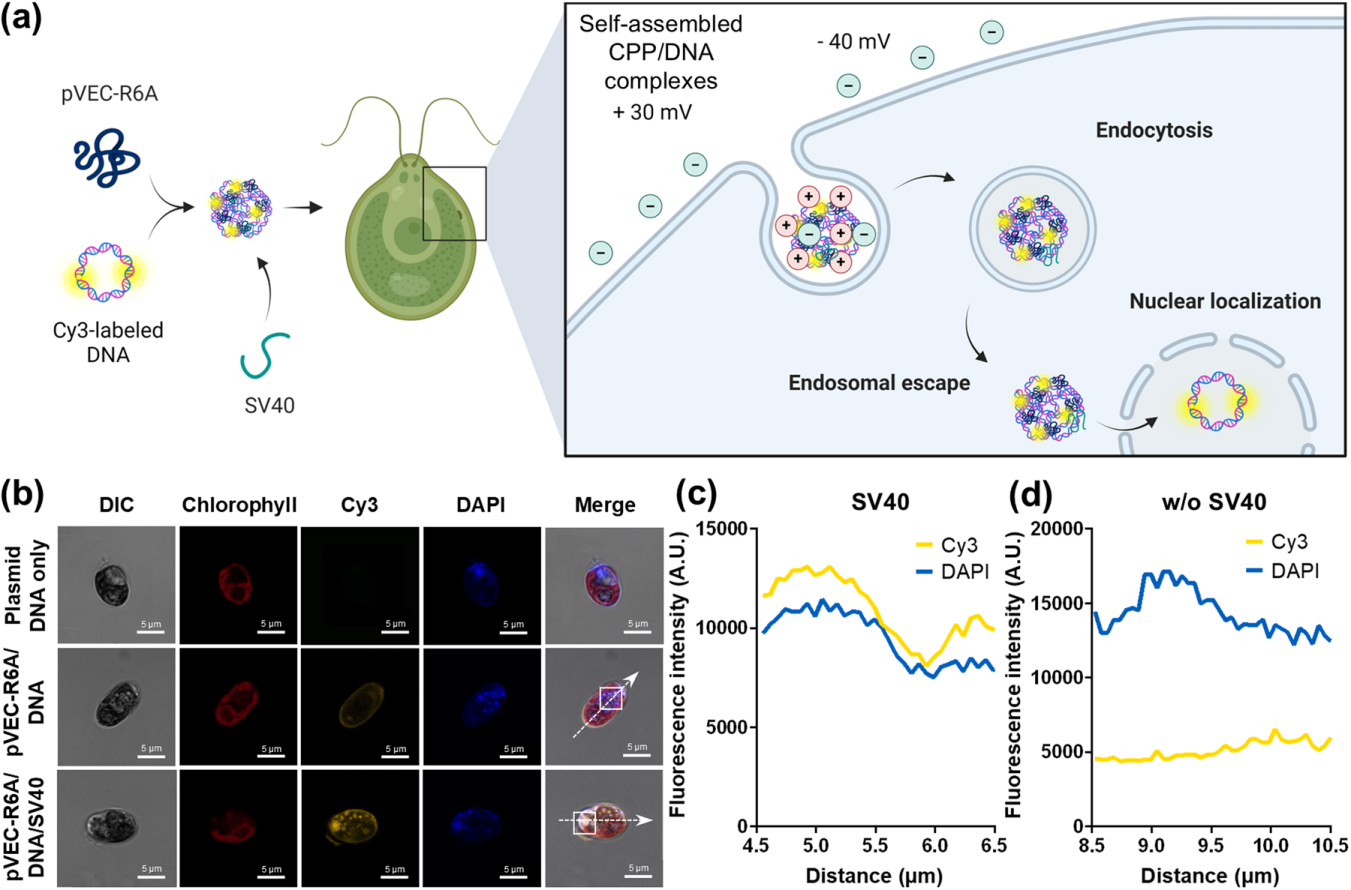
Nuclear localization of CPP/DNA/SV40 triple nanocomplexes. (a) Schematic illustration of the triple nanocomplex formation by pVEC‐R6A, Cy3 labeled‐DNA, and SV40 peptide, and the proposed cellular uptake mechanism. (b) Cellular internalization of Cy3‐labeled pChlamy4 in 4′,6‐diamidino‐2‐phenylindole (DAPI)‐stained microalgal cells after treatment with the triple nanocomplex. White arrows indicate the trajectories of Cy3 and DAPI fluorescence in (c) and (d).

To evaluate nuclear localization, we monitored the localization of plasmid DNA in the triple nanocomplex to determine its ability to colocalize with nuclear DNA. The triple nanocomplexes were formed by combining Cy3‐labeled plasmid DNA with pVEC‐R6A and SV40 peptides. Designed to target the *C. reinhardtii* cell membrane, which is relatively anionic, the nanocomplexes are capable of electrostatically interacting with the cell membrane before localizing in the cytoplasm (Li et al. 2022). In the cytoplasm, SV40 facilitates the transport of nanocomplex to the nucleus (Figure 5a). First, we evaluated the impact of SV40 on nuclear localization in microalgal cells. Cells treated with the triple nanocomplexes showed significant signal overlap between DAPI‐stained nuclei and Cy3‐labeled plasmid DNA, demonstrating efficient delivery of plasmid DNA into the nucleus (Figure 5b). Further analysis of fluorescence intensity confirmed this co‐localization, showing a strong correlation (*r* = 0.9287) between DAPI and Cy3 fluorescence signals when SV40 was present in the triple nanocomplexes (Figure 5c; Supporting Information S1: Figure S1a). In contrast, a poor correlation (*r* = −0.5704) was observed between DAPI and Cy3 signals in the absence of SV40, highlighting its critical role in facilitating nuclear transport (Figure 5d; Supporting Information S1: Figure S1). Overall, the introduction of SV40 significantly enhanced the nuclear transport and localization of plasmid DNA when combined with pVEC‐R6A, suggesting a promising strategy to overcome the nuclear membrane barrier in microalgae.

Despite the cationic nature of SV40, no complexation between SV40 and DNA was observed (Supporting Information S1: Figure S2). Although SV40 serves as a carrier across the nuclear membrane, its transfection efficiency remains relatively low (Jeong et al. 2021; van der Aa et al. 2005). Our findings suggest the potential of SV40 as an adjuvant in the formation of nanocomplexes with DNA and CPPs. Future research is needed to fully understand the interaction mechanisms in microalgae, focusing on the specific proteins that may interact with the triple nanocomplexes and the potential impacts on microalgal cell function.

Additionally, we investigated the cellular uptake mechanism of the triple nanocomplexes using wortmannin, an endocytosis inhibitor. This inhibitor reduced the normalized Cy3/chlorophyll *a* level by 50% compared to the control (Supporting Information S1: Figure S3), suggesting the involvement of endocytosis in the delivery of the triple nanocomplexes. Further studies should investigate other cellular uptake mechanisms to elucidate the multifaceted pathways involved in this process.

### 3.6 Microalgal Transformation With pVEC‐R6A/DNA/SV40 Triple Nanocomplex

Genetic transformation using the pVEC‐R6A/DNA/SV40 triple nanocomplex was evaluated in *C. reinhardtii* CC‐124, an algal strain with a cell wall consisting of hydroxyproline‐rich glycoproteins (Adair and Apt 1990). A transformation efficiency of 63.9 ± 39.5 transformants per µg DNA per 1.5 × 10^7^ initial cells was achieved. Several microalgal species, including *C. reinhardtii*, are sensitive to zeocin (Adair and Apt 1990; Liu et al. 2013; López‐Paz et al. 2017; Osorio et al. 2019). Therefore, the pChlamy4 vector, containing the zeocin resistance gene, was used to easily identify positive transformants on zeocin‐containing agar plates (Supporting Information S1: Figure S4). Numerous studies have demonstrated the ability of various CPPs to penetrate and deliver biological cargos, including small and large proteins, dsDNA, and dsRNA, into various microalgae (Gadamchetty et al. 2019; Hyman et al. 2012; Kang et al. 2017; Suresh and Kim 2013; Wei et al. 2015). A recent study highlighted the potential of CPPs in delivering ribonucleoprotein (Cas9/sgRNA complex) to *C. reinhardtii*, with or without cell walls, to knock out *Maa7* and *FKB12* genes (Kang et al. 2020). However, only one study reported CPP‐mediated genetic modification of microalgae in *C. vulgaris* (Gadamchetty et al. 2019). In that study, the highest transformation efficiency was 1.1 × 10^3^ ± 0.029 transformants per µg DNA when 2 × 10^5^ *C. vulgaris* cells were transfected with linear T‐DNA, mediated by TAT peptide (Gadamchetty et al. 2019). This high efficiency was achieved when cells were treated with 0.1% Triton X‐100, a non‐ionic surfactant, to permeabilize the cell wall. Without this pretreatment, the TAT peptide was unable to generate positive transformants. In comparison, conventional transformation methods such as *Agrobacterium‐*mediated transformation yielded a maximum efficiency of 14 transformants per 10^8^ cells with the cell‐walled *C. reinhardtii* CC125 wild‐type strain, and a higher efficiency of 33 transformants per 10^8^ cells when the cell wall‐deficient cw15 strain was used (Mini et al. 2018). On the other hand, the square‐wave electroporation method achieved 2–6 × 10^3^ transformants per µg DNA per 5 × 10^7^ cells for *C. reinhardtii* cell‐walled strains in a strain‐dependent manner (Wang et al. 2019). These results suggest that although CPP‐mediated transformation shows great promise, further investigation is needed to improve its efficiency and broaden its applications in the biotechnological field.

Further studies should explore targeting strategies for subcellular organelles, such as chloroplasts and mitochondria, along with the nucleus in microalgae. Recent advances in CPP technology have led to the development of targeted peptides for specific organelle localization, moving beyond previous studies that primarily focused on nuclear targeting (Thagun et al. 2019). For example, chloroplast‐targeting peptide sequences ligated to the C‐terminus of CPPs showed enhanced translocation to chloroplasts in plant cells (Thagun et al. 2022). In microalgae, effective targeting of both organelles and nuclei is crucial, as mitochondria and chloroplasts play significant roles in many vital processes, such as photosynthesis and metabolite production (Gomez‐Casati et al. 2022). Future studies should apply chloroplast‐ and mitochondria‐targeting peptides commonly used in plant research to microalgae and develop innovative gene delivery systems. These advancements could facilitate precise genetic modification of microalgae to improve their CO₂ reduction and value‐added compound production, ultimately aiding in the mitigation of climate change.

## 4 Conclusions

In this study, we developed a novel CPP/DNA/SV40 triple nanocomplex system that facilitates efficient nuclear translocation of plasmid DNA while maintaining a simple preparation procedure. Our evaluation of pVEC‐ORI and pVEC‐R6A showed that pVEC‐R6A significantly outperformed pVEC‐ORI in facilitating DNA uptake in microalgae. Furthermore, by incorporating the SV40 NLS peptide into the CPP/DNA nanocomplex, we successfully directed plasmid DNA into the nucleus and achieved successful genetic transformation in *C. reinhardtii*. Our results highlight the potential of CPP‐based systems as effective tools for microalgal genetic engineering and biotechnological advancement.

## Author Contributions

**Eun Jeong Sim:** formal analysis, writing – original draft. **Quynh‐Giao Tran:** validation, writing – review and editing. **Yu Rim Lee:** validation. **Trang Thi Le:** formal analysis. **Hyang Ran Yoon:** visualization. **Dong‐Yun Choi:** project administration. **Dae‐Hyun Cho:** data curation. **Jin‐Ho Yun:** data curation. **Hong Il Choi:** data curation. **Hee‐Sik Kim:** supervision, writing – review and editing. **Yong Jae Lee:** supervision, conceptualization, writing – review and editing. All authors provided feedback on the work, and reviewed and proofread the final version of the article.

## Supporting information

Pearson correlation coefficient for the degree of co‐localization between plasmid DNA and nuclei; Electrophoretic mobility shift assay (EMSA) to confirm the complex formation between SV40 NLS peptide and plasmid DNA; Quantification of fluorescence intensity to study the cellular uptake mechanism of nanocomplexes; Genetic transformation of *Chlamydomonas reinhardtii* mediated by triple nanocomplexes (DOCX).

## Data Availability

The data that support the findings of this study are available from the corresponding author upon reasonable request.
