# Supplementary material for: Cell‐Penetrating Peptide‐Based Triple Nanocomplex Enables Efficient Nuclear Gene Delivery in Chlamydomonas reinhardtii
Source: Biotechnol Bioeng. 2025 May 8;122(8):2218–27. doi: 10.1002/bit.29019 (PMC12235220; doi:10.1002/bit.29019)
Supplement: Supplementary file 1 — Pearson correlation coefficient for the degree of co‐localization between plasmid DNA and nuclei; Electrophoretic mobility shift assay (EMSA) to confirm the complex formation between SV40 NLS peptide and plasmid DNA; Quantification of fluorescence intensity to study the cellular uptake mechanism of nanocomplexes; Genetic transformation of Chlamydomonas reinhardtii mediated by triple nanocomplexes (DOCX). [file BIT-122-2218-s001.docx]

*Supplementary information for:*

**Cell-Penetrating Peptide-Based Triple Nanocomplex Enables Efficient Nuclear Gene Delivery in *Chlamydomonas reinhardtii***

Eun Jeong Sim^1,2‡^, Quynh-Giao Tran^1‡^, Yu Rim Lee^1^, Trang Thi Le^1,2^, Hyang Ran Yoon^3^, Dong-Yun Choi^1^, Dae-Hyun Cho^1^, Jin-Ho Yun^1,2^, Hong Il Choi^1,2^, Hee-Sik Kim^1,2*^, and Yong Jae Lee^1,2*^

^1^Cell Factory Research Center, Korea Research Institute of Bioscience and Biotechnology (KRIBB), Daejeon 34141, Republic of Korea

^2^Department of Environmental Biotechnology, KRIBB School of Biotechnology, University of Science and Technology (UST), Daejeon 34113, Republic of Korea

^3^Immunotherapy Convergence Research Center, KRIBB, Daejeon 34141, Republic of Korea

^*^Corresponding authors: leeyj@kribb.re.kr; hkim@kribb.re.kr

^‡^These authors contribute equally and share first authorship.


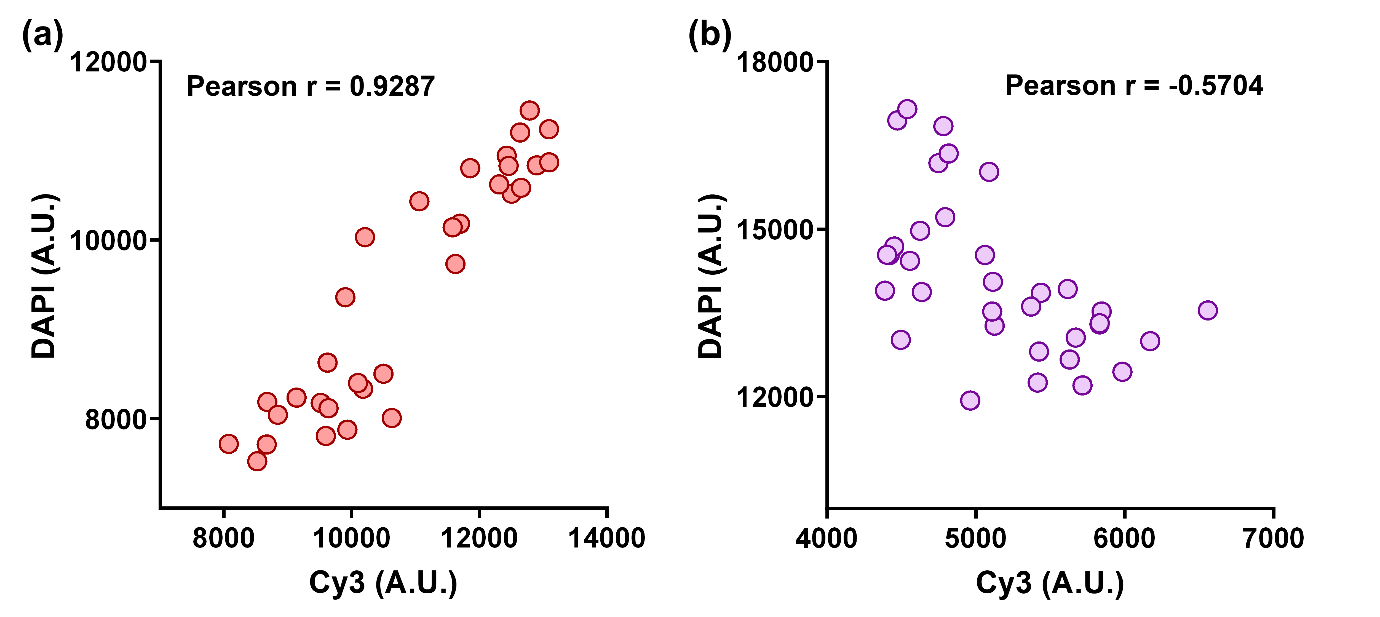


**Figure S1.** Pearson correlation coefficient as a measure of co-localization between plasmid DNA and nuclear DNA. Scatter plots showing the correlation between DAPI-stained nuclear DNA and Cy3-labeled plasmid DNA from **Figure 5c**, depicting complexes formed with plasmid DNA, pVEC-R6A, in the presence of SV40 (panel a) or from **Figure 5d**, depicting complexes formed with plasmid DNA, pVEC-R6A, in the absence of SV40 (panel b). The experimental conditions as described in **Figure 5** were applied. The number of XY pairs is 33 for panel a and 40 for panel b. Pearson r was calculated with 99% confidence.


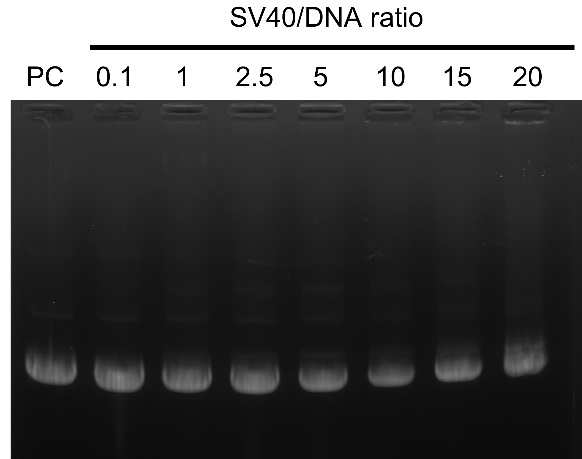


**Figure S2.** Confirmation of complex formation between SV40 NLS peptide and plasmid DNA. To explore the ability of SV40 to form complexes with plasmid DNA, a series of SV40/DNA ratios were tested, including 0.1:1, 1:1, 2.5:1, 5:1, 10:1, 15:1, and 20:1. A total of 0.5 μg of pChlamy4 vector was mixed with SV40 to obtain the corresponding ratios and incubated for 20 min. After incubation, samples were analyzed by 1% (w/v) agarose gel electrophoresis. The results indicated that SV40 itself did not bind to plasmid DNA. The positive control (PC) was the naked pChlamy4 plasmid vector.

**Figure S3.** Investigation of cellular uptake mechanism of triple nanocomplexes. *C. reinhardtii* cells were pretreated with the endocytosis inhibitor wortmannin. The pChlaymy4 vector was labeled with Cy3 and formed nanocomplexes with pVEC-R6A and SV40 NLS. Fluorescence intensity was quantified and expressed as relative units, normalized to the control (cells treated with TAP medium containing 5% dimethyl sulfoxide). Statistical significance is indicated by ***p* < 0.01.


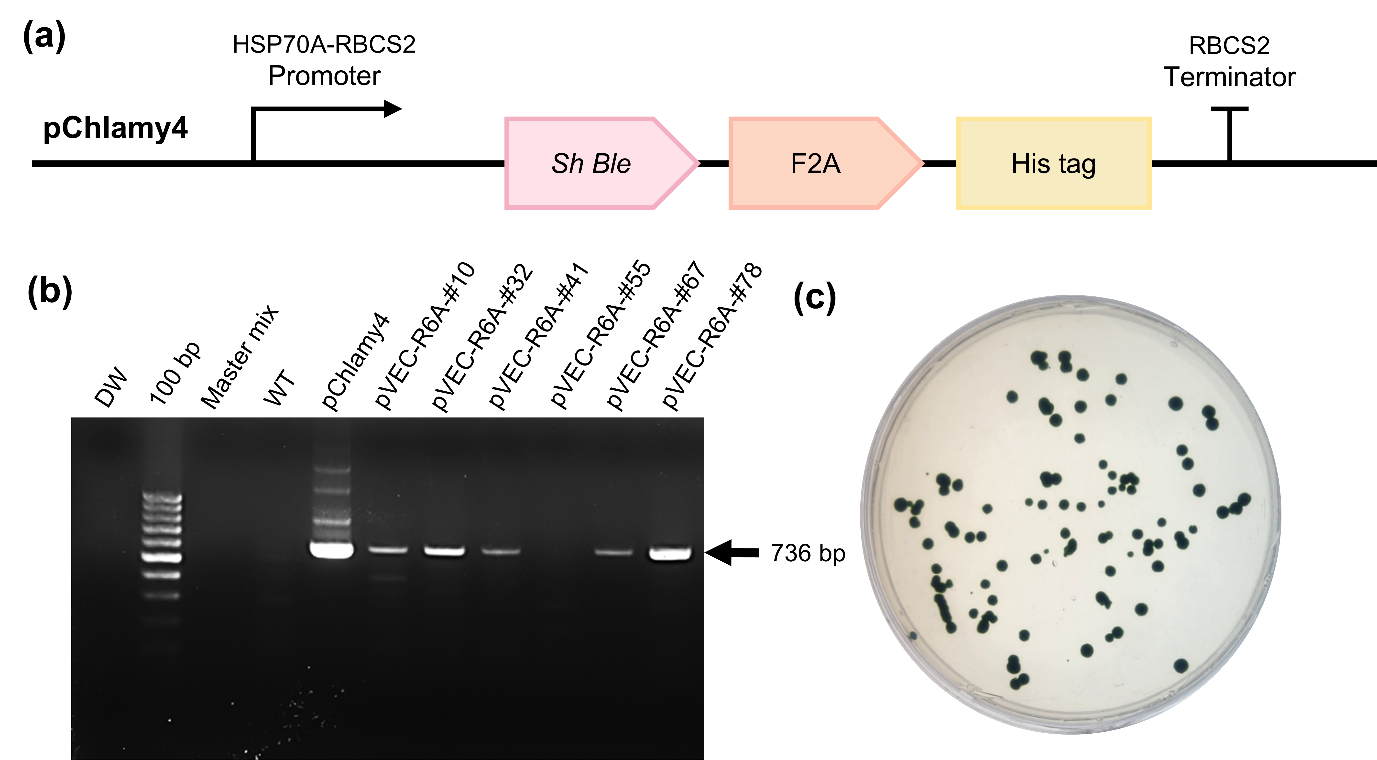


**Figure S4.** Genetic transformation of *Chlamydomonas reinhardtii* mediated by triple nanocomplexes. (a) Schematic diagram of the plasmid DNA used for transfection into *C. reinhardtii*. (b) Colony PCR analysis using primers specific for the zeocin resistance gene. Samples included: DW (nuclease-free water), 100 bp ladder, Master mix (PCR reaction without DNA), WT (untransformed *C. reinhardtii*), pChlamy4 (plasmid DNA), randomly selected zeocin-resistant colonies (pVEC-R6A #10, 32, 41, 55, 67, 78). (c) Zeocin-resistant colonies cultured for 20 days on TAP agar plates containing 5 ppm zeocin.
